# Relationship Between Change in Serum Uric Acid and Ischemic Stroke in Chinese Hypertensive Patients

**DOI:** 10.3389/fcvm.2021.717128

**Published:** 2021-09-21

**Authors:** Qiu-hong Tan, Lin Liu, Yu-qing Huang, Yu-ling Yu, Jia-yi Huang, Chao-lei Chen, Song-tao Tang, Ying-qing Feng

**Affiliations:** ^1^School of Biology and Biological Engineering, South China University of Technology, Guangzhou, China; ^2^Department of Cardiology, Guangdong Cardiovascular Institute, Guangdong Provincial People's Hospital, Guangdong Academy of Medical Sciences, Guangzhou, China; ^3^Department of Cardiology, Community Health Center of Liaobu County, Dongguan, China

**Keywords:** uric acid, variability, ischemic stroke, hypertension, epidemiology

## Abstract

**Background:** Limited studies focused on the association between serum uric acid (SUA) change with ischemic stroke, and their results remain controversial. The present study aimed to investigate the relationship between change in SUA with ischemic stroke among hypertensive patients.

**Method:** This was a retrospective cohort study. We recruited adult hypertensive patients who had two consecutive measurements of SUA levels from 2013 to 2014 and reported no history of stroke. Change in SUA was assessed as SUA concentration measured in 2014 minus SUA concentration in 2013. Multivariable Cox proportional hazards models were used to estimate adjusted hazard ratios (HRs) and 95% confidence intervals (CIs). The Kaplan–Meier analysis and log-rank test were performed to quantify the difference in cumulative event rate. Additionally, subgroup analysis and interaction tests were conducted to investigate heterogeneity.

**Results:** A total of 4,628 hypertensive patients were included, and 93 cases of ischemic stroke occurred during the mean follow-up time of 3.14 years. Participants were categorized into three groups according to their SUA change tertiles [low (SUA decrease substantially): <-32.6 μmol/L; middle (SUA stable): ≥-32.6 μmol/L, <40.2 μmol/L; high (SUA increase substantially): ≥40.2 μmol/L]. In the fully adjusted model, setting the SUA stable group as reference, participants in the SUA increase substantially group had a significantly elevated risk of ischemic stroke [HR (95% CI), 1.76 (1.01, 3.06), *P* = 0.0451], but for the SUA decrease substantially group, the hazard effect was insignificant [HR (95% CI), 1.31 (0.75, 2.28), *P* = 0.3353]. Age played an interactive role in the relationship between SUA change and ischemic stroke. Younger participants (age < 65 years) tended to have a higher risk of ischemic stroke when SUA increase substantially.

**Conclusion:** SUA increase substantially was significantly correlated with an elevated risk of ischemic stroke among patients with hypertension.

## Introduction

Stroke is a common cause of death and disability worldwide; approximately 87% is an ischemic subtype (1). In China, stroke ranks the first leading cause of mortality, and more than two million individuals suffer from new-onset annually (2), which imposes a substantial burden on public health systems. Therefore, the identification of stroke risk factors is vital for prevention and early detection. Diabetes (3), atrial fibrillation (4), and hypertension (5) are well-established risk factors of stroke. In addition, more and more evidence suggests that serum uric acid (SUA) level is implicated in stroke (6–8).

Uric acid (UA) is the final product of purine metabolism, catalyzed by the xanthine oxidase (XO) (9). Due to its powerful antioxidant capacity, UA plays an essential role in preventing damage of free radicals (10). It has been reported that both excessive and insufficient SUAs are closely related to cardiovascular and cerebrovascular diseases (11, 12). Recently, epidemiological studies showed that the fluctuation of metabolic parameters, including body weight (13), serum copper (14), and plasma triglyceride (15), increased the risk of cardiovascular events. Although some studies found that large variation of SUA often resulted in adverse events (16, 17), limited studies focused on the relationship between changes in SUA and stroke, and their results remained conflicts. Research based on subjects who underwent percutaneous coronary intervention suggested that abrupt change in SUA was significantly correlated with an elevated risk of ischemic stroke (18). In contrast, the other two cohort studies that enrolled patients with a history of cardiovascular disease did not find a similar pattern (19, 20). Therefore, we sought to examine the association between SUA change and ischemic stroke among the hypertensive population.

## Materials and Methods

### Study Population

In the current retrospective cohort study, all participants were sourced from an annual physical examination program carried out in Liaobu town, Dongguan, China. In brief, SUA data were gathered from 2013 to 2014 to assess the magnitude of SUA changes, and follow-up data were collected between 2014 and 2017.

**Figure 1:**
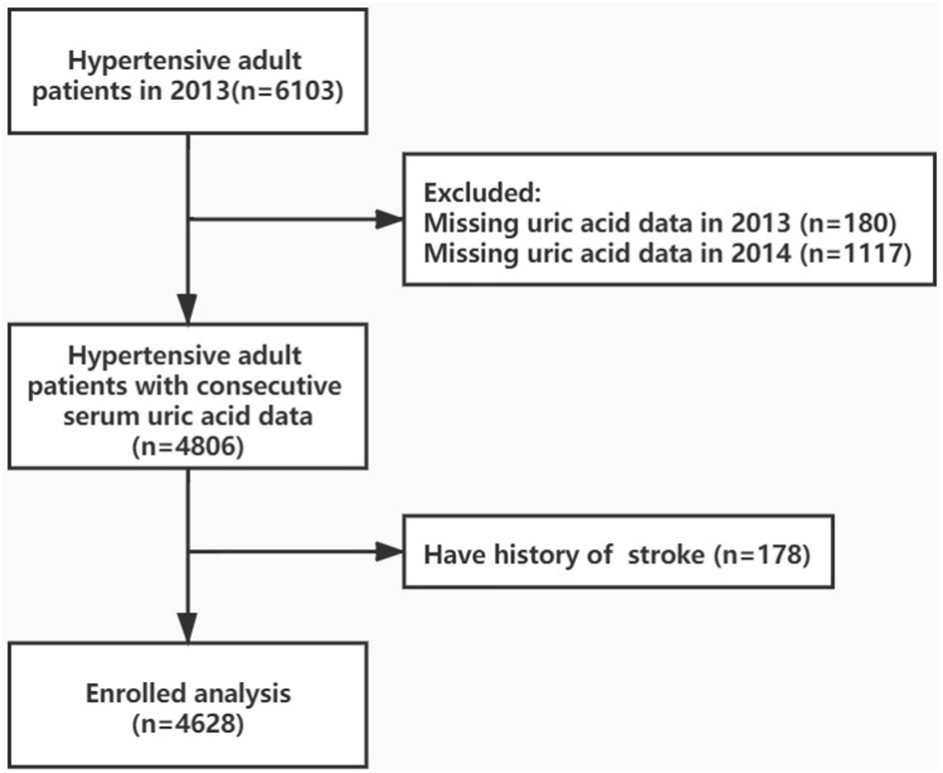
Research flow chart.

The original cohort included 6,103 adult hypertensive participants in 2013. We excluded those participants who missed the SUA data in 2013–2014 (*n* = 180 and 1,117, respectively) or have a history of stroke (*n* = 178). Finally, 4,628 participants were enrolled in the present study (Figure 1). The present research conformed to the Helsinki declaration, and ethical approval was obtained by the institutional medical ethics committee of Guangdong Provincial People's Hospital.

### Measurements of Serum Uric Acid Change

Concentrations of SUA were determined annually during the 2013–2014 annual physical examination program. Fasting blood samples were drawn after 8–10 h of overnight fasting. After that, the samples were centrifuged at 3,500 rpm for 15 min to obtain a serum layer for analysis. The concentration of SUA was measured using an automatic biochemical analyzer (Hitachi 7170A). In the present study, SUA change was defined as SUA concentration change from 2013 to 2014 (SUA change = SUA_2014_ – SUA_2013_).

### Measurements of Covariates

Data of demographic information (age and sex), disease history (hypertension, stroke, diabetes, and coronary heart disease), use of meditation (antihypertensive drugs, UA-lowering drugs, lipid-lowering drugs, diuretics, antiplatelet drugs, and hypoglycemic agents), lifestyle (physical activity, smoking, and drinking) were collected through face-to-face questionnaire interview. Physical measurements included height, weight, neck circumference, systolic blood pressure (SBP), and diastolic blood pressure (DBP). Measurements of biomarkers, which included triglyceride (TG), high-density lipoprotein cholesterol (HDL-C), low-density lipoprotein cholesterol (LDL-C), and white blood cell (WBC), were measured in the central laboratory of Liaobu. All processes were conducted by trained medical staff and adhered to standardized procedures and protocols. Body mass index (BMI) was calculated from height and weight. Hypertension was defined as blood pressure ≥ 140/90 mmHg, a self-reported history of hypertension, or current taking antihypertension drugs (21). Diabetes was defined as fasting blood glucose e ≥ 126 mg/dl, a self-reported history of diabetes, hemoglobin A1c ≥ 6.5%, or currently using a hypoglycemic drug (22). The estimated glomerular filtration rate (eGFR, ml/min/1.73 m^2^) was calculated using the abbreviated equation developed by the Modification of Diet in Renal Disease study (23). Data of covariates were measured in 2014.

### Outcome

The endpoint of the present study was the first occurrence of ischemic stroke during the follow-up period. In brief, the clinical diagnosis of stroke was adjudicated by qualified professional neurologists, based on neurological examination, stroke scale assessment, laboratory tests, and presence of acute infarction on the cranial computed tomography or magnetic resonance imaging (24). All ischemic stroke events were obtained from the local medical insurance system of the medical insurance bureau, and any participants who had no medical records available were followed up by in-person interviews or telephone contacts until December 31, 2017.

### Statistical Analysis

Continuous values are expressed as means ± standard deviations or as median (interquartile range), according to the normality of the distribution. Categorical variables were expressed as frequency and percentage. Differences between groups were assessed with a one-way analysis of variance and chi-square. If a continuous variable was lack of normality, the Kruskal-Wallis and Wilcoxon rank-sum test was considered. Participants were categorized into three groups according to their SUA change tertiles [low (SUA decrease substantially): <-32.6 μmol/L; middle (SUA stable): ≥-32.6 μmol/L, <40.2 μmol/L; high (SUA increase substantially): ≥40.2 μmol/L]. Hazard ratios (HRs) and 95% confidence intervals (CIs) for ischemic stroke were estimated by the Cox proportional hazard regression models. Model I was a non-adjusted model; model II was adjusted for age and sex; model III was adjusted for age, sex, SBP, DBP, BMI, neck circumference, TG, HDL-C, LDL-C, eGFR, WBC, baseline SUA, diabetes, coronary heart disease, physical activity, smoking, drinking, diuretics, antihypertensive drugs, hypoglycemic agents, lipid-lowering drugs, antiplatelet drugs, and UA-lowering drugs. Cumulative event rates for each group were expressed using the Kaplan–Meier method and compared using the log-rank test. Subgroup analysis and tests for interactions were performed, including age (<65 or ≥65 years), sex (male or female), smoking status (yes or no), baseline SUA concentration (<360 or ≥360 μmol/L), and diabetes (yes or no). Significance was assigned as *P* < 0.05 (two-sided). All statistical analyses were performed using R version 3.4.3 (R Foundation for Statistical Computing, Vienna, Austria) and Empower (R) (www.empowerstats.com; X&Y solutions, Inc., Boston, MA, USA).

## Results

### Baseline Characteristics

Participants' baseline characteristics are presented in Table 1. Of the total 4,628 participants, the mean age was 62.13 ± 13.02 years, and 2,524 (54.54%) were female. Over the mean follow-up time of 3.14 years, 93 participants experienced new-onset ischemic stroke. We observed a significant difference in age, DBP, neck circumference, TG, eGFR, baseline SUA, diabetes, and the use of antihypertensive drugs, hypoglycemic agents, and UA-lowering drugs among groups according to SUA change levels (all *P* < 0.05).

**Table 1:** Baseline characteristics of participants.

	**Total**	**SUA decrease**	**SUA stable**	**SUA increase**	***P*-value**
Number	4,628	1,543	1,542	1,543	
Age, year	62.13 ± 13.02	62.72 ± 12.99	61.46 ± 13.34	62.21 ± 12.69	0.025
Sex (*n*, %)					0.187
Male	2,104 (45.46%)	719 (46.60%)	672 (43.58%)	713 (46.21%)	
Female	2,524 (54.54%)	824 (53.40%)	870 (56.42%)	830 (53.79%)	
SBP, mmHg	141.06 ± 18.26	141.87 ± 18.26	140.93 ± 18.53	140.39 ± 17.96	0.073
DBP, mmHg	82.13 ± 11.62	82.15 ± 11.69	82.72 ± 11.57	81.51 ± 11.56	0.016
BMI, kg/m^2^	25.57 ± 3.87	25.54 ± 3.92	25.44 ± 3.83	25.73 ± 3.86	0.114
Neck circumference, cm (median, IQR)^*^	35.00 (33.00–37.50)	35.00 (33.00–37.50)	35.00 (33.00–37.20)	35.00 (33.00–38.00)	0.025
TG, mg/dl, (median, IQR)^*^	127.80 (90.57–185.93)	127.30 (90.70–181.10)	122.05 (86.60–178.02)	135.90 (95.75–201.65)	<0.001
HDL-C, mg/dl	56.69 ± 13.80	56.66 ± 12.76	57.14 ± 15.15	56.26 ± 13.38	0.21
LDL-C, mg/dl	119.55 ± 32.69	119.30 ± 32.50	119.89 ± 33.37	119.47 ± 32.20	0.878
eGFR, ml/min/1.73 m^2^	101.76 ± 28.66	103.99 ± 28.90	104.66 ± 28.42	96.64 ± 27.97	<0.001
WBC, 10∧9/L	7.50 ± 1.99	7.52 ± 1.89	7.41 ± 2.15	7.57 ± 1.91	0.072
Baseline SUA, μmol/L	408.09 ± 106.57	377.12 ± 93.70	384.95 ± 95.23	462.20 ± 108.68	<0.001
Diabetes (*n*, %)	1092 (23.60%)	362 (23.46%)	325 (21.08%)	405 (26.25%)	0.003
Coronary heart disease (*n*, %)	110 (2.38%)	35 (2.27%)	33 (2.14%)	42 (2.72%)	0.535
Physical activity (*n*, %)					0.053
Everyday	2184 (47.19%)	733 (47.50%)	734 (47.60%)	717 (46.47%)	
Often	286 (6.18%)	105 (6.80%)	106 (6.87%)	75 (4.86%)	
Occasionally	304 (6.57%)	100 (6.48%)	111 (7.20%)	93 (6.03%)	
Never	1854 (40.06%)	605 (39.21%)	591 (38.33%)	658 (42.64%)	
Smoking (*n*, %)	1180 (25.50%)	411 (26.64%)	370 (23.99%)	399 (25.86%)	0.224
Drinking (*n*, %)	674 (14.56%)	209 (13.55%)	216 (14.01%)	249 (16.14%)	0.093
Diuretics (*n*, %)	446 (9.64%)	132 (8.55%)	145 (9.40%)	169 (10.95%)	0.073
Antihypertensive drugs (*n*, %)	3604 (77.87%)	1236 (80.10%)	1178 (76.39%)	1190 (77.12%)	0.031
Hypoglycemic agents (*n*, %)	732 (15.82%)	250 (16.20%)	210 (13.62%)	272 (17.63%)	0.008
Lipid-lowering drugs (*n*, %)	1,089 (23.53%)	382 (24.76%)	352 (22.83%)	355 (23.01%)	0.378
Antiplatelet drugs (*n*, %)	672 (14.52%)	207 (13.42%)	221 (14.33%)	244 (15.81%)	0.162
Uric acid-lowering drugs (*n*, %)	55 (1.19%)	28 (1.81%)	8 (0.52%)	19 (1.23%)	0.004
Ischemic stroke (*n*, %)	93 (2.01%)	31 (2.01%)	23 (1.49%)	39 (2.53%)	0.122

*Data are reported as mean ± standard deviation unless otherwise stated. SUA, serum uric acid; SBP, systolic blood pressure; DBP, diastolic blood pressure; BMI, body mass index; TG, triglycerides; HDL-C, high-density lipoprotein cholesterol; LDL-C, low-density lipoprotein cholesterol; eGFR, estimated glomerular filtration rate; WBC, white blood cell; IQR, interquartile range*.

* *Values are reported as median (interquartile range) due to non-normality*.

### Association Between Serum Uric Acid Change and Ischemic Stroke

As summarized in Table 2, Cox regression analyses were performed to investigate the association of SUA change with ischemic stroke. In model III, when SUA changes were expressed as continuous variables, a non-significantly elevated risk for ischemic stroke in the company with the increment of SUA change was noted [HR (95% CI), 1.10 (0.88, 1.39) per SD increment, *P* = 0.4069]. When SUA change was expressed as a categorical variable, participants in the SUA increase substantially group had a higher risk of ischemic stroke compared with those in the SUA stable group, and statistically significant differences were marked [HR (95% CI), 1.76 (1.01, 3.06), *P* = 0.0451], but for the SUA decrease substantially group, the hazard effect was noted but insignificant [HR (95% CI), 1.31 (0.75, 2.28), *P* = 0.3353].

**Table 2:** Relationship between SUA variability with ischemic stroke.

	**Event rate/person-years**	**Model I** **HR (95%CI) *P*-value**	**Model II** **HR (95%CI) *P*-value**	**Model III** **HR (95%CI) *P*-value**
SUA change per SD increment		1.11 (0.91, 1.36) 0.3040	1.11 (0.91, 1.35) 0.3178	1.10 (0.88, 1.39) 0.4069
SUA stable	0.0048 (23/4842)	Reference	Reference	Reference
SUA decrease substantially	0.0064 (31/4830)	1.35 (0.79, 2.31) 0.2787	1.30 (0.76, 2.24) 0.3342	1.31 (0.75, 2.28) 0.3353
SUA increase substantially	0.008 (39/4845)	1.70 (1.01, 2.84) 0.0439	1.67 (1.00, 2.80) 0.0500	1.76 (1.01, 3.06) 0.0451

*Model I adjust for none. Model II adjust for Age and sex. Model III adjust for: Age, sex, SBP, DBP, BMI, neck circumference, TG, HDL-C, LDL-C, eGFR, WBC, baseline SUA, diabetes, coronary heart disease, physical activity, smoking, drinking, diuretics, antihypertensive drugs, hypoglycemic agents, lipid-lowering drugs, antiplatelet drugs, and uric acid-lowering drugs. HR, hazard ratios; CI, confidence intervals; SUA, serum uric acid*.

**Figure 2:**
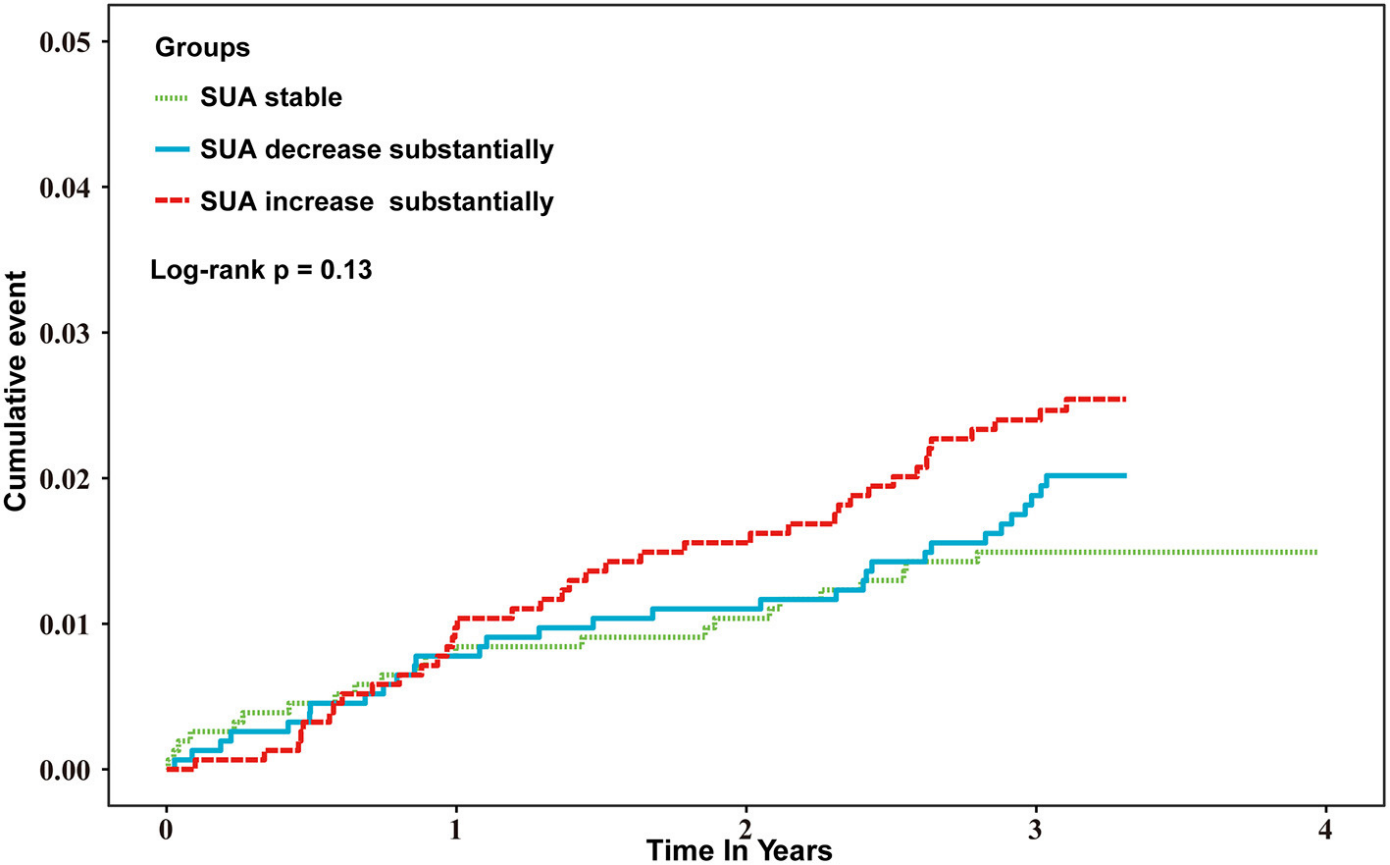
Kaplan–Meier estimates of ischemic stroke-cumulative event by SUA change.

As depicted in Figure 2, the cumulative incidence of ischemic stroke was higher in the SUA increase substantially group, but the differences between groups were not significant (*P* = 0.13).

### Subgroup Analysis

Subgroup analysis investigating the effect of age, sex, smoking status, baseline SUA concentration, and diabetes is shown in Table 3. There was an interaction for age and SUA change (*P* for interaction = 0.0185). Participants aged < 65 years had a higher risk of ischemic stroke [HR (95% CI), 1.76 (1.01, 3.06), *P* = 0.0451] with SUA increase substantially.

**Table 3:** Subgroup analysis of SUA change with ischemic stroke.

	**Event rate/person-years**	**SUA stable**	**SUA decrease substantially** **HR (95%CI) *P*-value**	**SUA increase substantially** **HR (95%CI) *P*-value**	**P-interaction**
Age, year					0.0185
<65	0.0049 (40/8173)	Reference	1.21 (0.44, 3.31) 0.7087	3.18 (1.29, 7.85) 0.0120	
≥65	0.0083 (53/6359)	Reference	1.44 (0.73, 2.82) 0.2905	1.05 (0.50, 2.24) 0.8911	
Sex					0.3455
Male	0.0061 (40/6607)	Reference	1.09 (0.46, 2.61) 0.8386	2.46 (1.05, 5.73) 0.0377	
Female	0.0067 (53/7900)	Reference	1.58 (0.76, 3.28) 0.2188	1.59 (0.74, 3.41) 0.2312	
Smoking					0.3564
No	0.0057 (62/10827)	Reference	1.72 (0.86, 3.44) 0.1233	1.88 (0.92, 3.85) 0.0819	
Yes	0.0084 (31/3693)	Reference	0.84 (0.32, 2.21) 0.7181	1.88 (0.74, 4.79) 0.1879	
Baseline SUA, μmol/L					0.4150
<360	0.0053 (27/5099)	Reference	2.09 (0.83, 5.26) 0.1189	2.45 (0.75, 8.03) 0.1388	
≥360	0.007 (66/9417)	Reference	0.97 (0.47, 1.99) 0.9299	1.63 (0.88, 3.02) 0.1171	
Diabetes					0.08
No	0.0059 (66/11103)	Reference	1.04 (0.56, 1.93) 0.9077	1.26 (0.66, 2.40) 0.4787	
Yes	0.0079 (27/3407)	Reference	4.36 (0.92, 20.58) 0.0630	6.66 (1.45, 30.64) 0.0149	

*Adjust for age, sex, SBP, DBP, BMI, neck circumference, TG, HDL-C, LDL-C, eGFR, WBC, baseline SUA, diabetes, coronary heart disease, physical activity, smoking, drinking, diuretics, antihypertensive drugs, hypoglycemic agents, lipid-lowering drugs, antiplatelet drugs, and uric acid-lowering drugs except for subgroup variable itself. HR, hazard ratios; CI, confidence intervals; SUA, serum uric acid*.

## Discussion

In the present study, we found that a marked increase in SUA from baseline to year 1 was associated with a significantly elevated risk of ischemic stroke among patients with hypertension. In addition, it was also noted that a marked decrease in SUA correlated with an elevated risk of ischemic stroke but not significant. Moreover, we found that age played an interactive role in the relationship between SUA change and ischemic stroke. Younger participants (age < 65 years) tended to have a higher risk of ischemic stroke when SUA increase substantially.

Our results were consistent with some previous researches. A retrospective cohort study in Taipei reported that higher SUA variability was related to ischemic stroke in patients with coronary artery disease who received the percutaneous coronary intervention (18). Observation from Middelheim Interdisciplinary Stroke Study indicated that rapid change in SUA concentration was associated with poor prognosis of stroke (25). In addition, a cohort study conducted in Kailuan found that prominent increases or decreases in SUA correlated with higher all-cause mortality risk (26). However, results from several other studies did not correlate in accordance with ours. A population-based study reported no significant correlation between SUA increase ≥ 0.06 mmol/L and stroke in participants with isolated systolic hypertension (20). The Israeli Ischemic Heart Disease Project demonstrated that higher variability of SUA did not relate to stroke mortality (27). This inconsistency may be due to several reasons. First is the different adjustments. Compared with previous research, our study adjusted more confounding factors that were found to be associated with ischemic stroke, including lifestyle, prescription, and biomarkers (28). Second, differences in race, underlying disease, course of the disease, and follow-up time may contribute to heterogeneity in outcomes.

Substantial increase or decrease of SUA reflects individuals had been exposed to an excessive or deficient concentration of SUA and even underwent metabolic disorders. Most prior research found a U- or J-shaped association between SUA and stroke (29–31), suggested detrimental effects of extrema level of SUA. Although the exact mechanism of SUA change and ischemic stroke remains unclear, some possible interpretations have been found. First, it is extensively investigated that SUA acts as a mediator in oxidative stress, endothelial function, and renin–angiotensin system; therefore, abrupt change of SUA may disturb physiological functions of important tissues and finally results in thrombus formation, aggravation of hypertension, and eventually ischemic stroke (32–34). Second, a large increase in SUA may indicate elevated XO activity (35), which has been reported to generate superoxide and play an important role in the pathogenesis of stroke (36). In the animal model of spontaneously hypertensive rats, it was suggested that increased activity of XO accounts for major sources of reactive oxygen species and may contribute to hypertensive target organ damage (37, 38). Third, SUA possessed potent antioxidant and anti-inflammation effects (10). Thus, a sharp decrease of SUA could prolong the inflammatory process due to acute gout flares (19, 39), signifying a decline of antioxidant capacity (40). Moreover, it has been reported that SUA plays a role in neuroprotection after stroke (41). Hence, excessively low SUA may fail to provide neuroprotection against ischemic stroke injuries. Forth, one of the urate transporter-coding genes, SLC2A9, has been reported to associate with an increased risk of cardiovascular and cerebrovascular events (42). Therefore, for the management of UA in hypertensive individuals, dosing strategies of “start-low and go-slow” and close monitoring of SUA level should be established (43).

Several limitations exist in our study. First, our research was a retrospective cohort study that could not demonstrate causality of SUA change and ischemic stroke. Second, this study was a single-center study and enrolled hypertensive individuals only; thus, the results only apply to our study population, and whether it applied to other populations remains to be tested. Because of the short follow-up time and low event rates, statistical power may be diminished. Third, ischemic stroke is a complex and multifactorial disease. Although we have adjusted for some potential confounding factors, the possibility of residual confounding may still exist. Forth, a difference was found in some baseline characteristics of those included and excluded (Supplementary Table 1). Excluded individuals prone to be older, have a lower level of DBP, BMI, LDL-C, and eGFR, a higher proportion of males, and more likely to have hypoglycemic agents, lipid-lowering drugs, and antiplatelet drugs; thus, selection bias could not be inevitable in our analysis.

In conclusion, SUA increase substantially is significantly associated with a higher risk of ischemic stroke in hypertensive, especially for patients younger than 65 years.

## Data Availability Statement

The original contributions presented in the study are included in the article/Supplementary Material, further inquiries can be directed to the corresponding author/s.

## Ethics Statement

The studies involving human participants were reviewed and approved by Institutional Medical Ethical Committee of the Guangdong Provincial People's Hospital, Guangzhou, China. The patients/participants provided their written informed consent to participate in this study.

## Author Contributions

Y-qF: conceptualization. Q-hT, LL, and Y-qH: investigation. Q-hT: writing—original draft preparation. Q-hT, LL, Y-qH, Y-lY, J-yH, and C-lC: writing—review and editing. Y-qH, LL, S-tT, and Y-qF: data collection. Y-qF and S-tT: supervision. All authors have read and agreed to the published version of the manuscript.

## Funding

This research was supported by the Science and Technology Plan Program of Guangzhou (No. 201803040012), the Key Area R&D Program of Guangdong Province (No. 2019B020227005), Guangdong Provincial People's Hospital Clinical Research Fund (Y012018085), the Fundamental and Applied Basic Research Foundation Project of Guangdong Province (2020A1515010738), the Climbing Plan of Guangdong Provincial People's Hospital (DFJH2020022) and Guangdong Provincial Clinical Research Center for Cardiovascular disease (2020B1111170011).

## Conflict of Interest

The authors declare that the research was conducted in the absence of any commercial or financial relationships that could be construed as a potential conflict of interest.

## Publisher's Note

All claims expressed in this article are solely those of the authors and do not necessarily represent those of their affiliated organizations, or those of the publisher, the editors and the reviewers. Any product that may be evaluated in this article, or claim that may be made by its manufacturer, is not guaranteed or endorsed by the publisher.
