# Supplementary material for: Relationship Between Change in Serum Uric Acid and Ischemic Stroke in Chinese Hypertensive Patients
Source: Front Cardiovasc Med. 2021 Sep 21;8:717128. doi: 10.3389/fcvm.2021.717128 (PMC8490735; doi:10.3389/fcvm.2021.717128)
Supplement: Supplementary file 1 [file Table_1.DOC]

**Supplementary Table 1** Baseline Characteristics of Participants and Nonparticipants

|  | **Participants** | **Nonparticipants** | P-value |
| --- | --- | --- | --- |
| Number | 4628 | 1475 |  |
| SUA, umol/L | 408.09 ± 106.57 | 404.06 ± 100.33 | 0.557 |
| Age, year | 62.13 ± 13.02 | 63.73 ± 15.81 | <0.001 |
| SBP, mmHg | 141.06 ± 18.26 | 142.57 ± 21.66 | 0.108 |
| DBP, mmHg | 82.13 ± 11.62 | 79.20 ± 11.93 | <0.001 |
| BMI, kg/m^2^ | 25.57 ± 3.87 | 24.97 ± 3.94 | 0.003 |
| Neck circumference, cm (median, IQR)* | 35.00 (33.00-37.50) | 35.00 (33.00-37.00) | 0.571 |
| WBC, 10^9/L | 7.50 ± 1.99 | 7.59 ± 2.60 | 0.449 |
| TG, mg/dL (median, IQR)* | 127.80 (90.57-185.93) | 129.45 (86.35-195.55) | 0.970 |
| LDL-C, mg/dL | 119.55 ± 32.69 | 113.72 ± 38.05 | 0.006 |
| HDL-C, mg/dL | 56.69 ± 13.80 | 56.87 ± 17.88 | 0.837 |
| eGFR, mL/min/1.73 m2 | 101.76 ± 28.66 | 92.15 ± 28.09 | <0.001 |
| Sex (n, %) |  |  | 0.034 |
| Male | 2104 (45.46%) | 712 (48.63%) |  |
| Female | 2524 (54.54%) | 752 (51.37%) |  |
| Diabetes (n, %) | 1092 (23.60%) | 118 (27.76%) | 0.054 |
| Coronary heart disease (n, %) | 110 (2.38%) | 14 (3.29%) | 0.242 |
| Physical activity (n, %) |  |  | 0.101 |
| Everyday | 2184 (47.19%) | 198 (46.70%) |  |
| Often | 286 (6.18%) | 22 (5.19%) |  |
| Occasionally | 304 (6.57%) | 17 (4.01%) |  |
| Never | 1854 (40.06%) | 187 (44.10%) |  |
| Smoking (n, %) | 1180 (25.50%) | 108 (25.47%) | 0.991 |
| Drinking (n, %) | 674 (14.56%) | 54 (12.74%) | 0.305 |
| Diuretics (n, %) | 446 (9.64%) | 37 (8.71%) | 0.532 |
| Antihypertensive drugs (n, %) | 3604 (77.87%) | 339 (79.76%) | 0.368 |
| Hypoglycemic agents (n, %) | 732 (15.82%) | 88 (20.71%) | 0.009 |
| Lipid-lowering drugs (n, %) | 1089 (23.53%) | 123 (28.94%) | 0.012 |
| Antiplatelet drugs (n, %) | 672 (14.52%) | 99 (23.29%) | <0.001 |
| Uric acid-lowering drugs (n, %) | 55 (1.19%) | 4 (0.94%) | 0.65 |

Data are reported as mean ± standard deviation unless otherwise stated.

SUA, serum uric acid; SBP, systolic blood pressure; DBP, diastolic blood pressure; BMI, body mass index; TG, triglycerides; HDL-C, high-density lipoprotein cholesterol; LDL-C, low-density lipoprotein cholesterol; eGFR, estimate glomerular filtration rate; WBC, white blood cell.

*Values are reported as median (IQR) due to nonnormality
